# The impact and utilisation of patient-initiated follow-ups (PIFU) in the elective upper-limb clinic at secondary care

**DOI:** 10.1308/rcsann.2025.0073

**Published:** 2025-10-21

**Authors:** R Naskar, V Garg, E Middleton, R Moverley

**Affiliations:** University Hospitals Dorset, UK

**Keywords:** Follow-up, Clinics, Shoulder, Elbow

## Abstract

**Background:**

Musculoskeletal (MSK) conditions affect over 20 million people in the UK. Follow-up appointments account for nearly two-thirds of all outpatient activity. Patient-initiated follow-up (PIFU) allows patients to request follow-up appointments based on clinical need, aiming to reduce unnecessary reviews and optimise service use.

This study evaluated the impact and utilisation of PIFU in a secondary care Elective Upper Limb (Shoulder and Elbow) Clinic, focusing on patient engagement, re-presentation rates, and suitability across common upper limb conditions.

**Methods:**

A retrospective service evaluation was conducted over 18 months. Patients discharged under the PIFU pathway were included if they had at least six months’ follow-up opportunity. Data collected included diagnosis, treatments received, re-presentation rates and outcomes. Descriptive analysis was used to assess patterns and clinical implications.

**Results:**

Out of 353 consultations, 81 patients (23%) were discharged to PIFU. Among these, 62% were first-time referrals discharged at their initial appointment. Conditions most suitable were rotator cuff-related pain (33%), early glenohumeral arthritis (19%) and adhesive capsulitis (10%). Within six months, only 18% of PIFU patients re-accessed care – just 4.2% of the total cohort. One-third of returning patients required surgery, while the remainder were managed nonoperatively and re-entered the PIFU pathway. PIFU freed approximately 20% of follow-up slots.

**Conclusions:**

PIFU is a safe and effective follow-up model for stable or early-stage shoulder and elbow conditions, reducing outpatient burden, supporting patient autonomy and preserving clinic capacity without compromising care quality. Broader implementation, supported by patient education and structured feedback, may enhance sustainable delivery of orthopaedic services.

## Introduction

The landscape of musculoskeletal (MSK) health in the UK is undergoing a significant transformation. With over 20.3 million individuals affected by conditions such as arthritis and back pain, MSK disorders now account for 21% of the nation’s years lived with disability.^1^ As the prevalence of these conditions increases, healthcare systems are under growing pressure to optimise outpatient services. Currently, follow-up appointments make up nearly two-thirds of all outpatient visits, which not only strains clinical resources but also limits access for new patients.^2^

In response, Patient-Initiated Follow-Up (PIFU) has emerged as an innovative approach to enhance the efficiency of outpatient care. Under PIFU, patients can schedule follow-up appointments based on their symptoms, rather than adhering to a fixed schedule for routine check-ups. This empowers patients, improves autonomy and eases the burden on secondary care services. Research has shown that PIFU models can reduce hospital reviews by up to 38% over six years, without compromising patient satisfaction.^3^ Moreover, the economic benefits are evident, as PIFU reduces healthcare expenses by cutting down on unnecessary hospital visits and travel costs for patients.

Despite its potential, there is limited research on the implementation of PIFU in Orthopaedic Shoulder and Elbow Clinics. This study aims to fill that gap by evaluating how PIFU is used in the Shoulder and Elbow Clinic, with a focus on patient engagement and return rates. We hope to determine whether PIFU can enhance care delivery, reduce outpatient workload and maintain clinical effectiveness in orthopaedic settings.

## Methodology

### Study design

This retrospective service evaluation was conducted in the Elective Shoulder and Elbow Clinic at a secondary care NHS Trust. Before data collection, approval was obtained from the Clinical Governance Department. The study aimed to assess the impact and utilisation of PIFU pathways over an 18-month period.

### Patient selection

Patients eligible for inclusion were those discharged under the PIFU pathway with a minimum of six months’ follow-up opportunity. The conditions reviewed included common upper limb issues such as rotator cuff tendinopathy, adhesive capsulitis, impingement syndrome and mild-to-moderate osteoarthritis. Postoperative patients and patients with conditions requiring ongoing surveillance, those with cognitive impairments or those facing communication barriers were excluded.

### PIFU intervention

In our clinic, the PIFU pathway was employed both as an alternative to routine follow-up for patients with stable or self-limiting conditions, and as a means of providing flexible, patient-led access to care following interventions where clinical response times can vary, such as ultrasound-guided injections. This approach allowed patients to return for further review based on their individual recovery trajectory, rather than adhering to fixed follow-up schedules.

At the point of discharge, patients selected for the PIFU pathway received both verbal and written instructions outlining how to re-access the clinic if needed. This included a direct contact number for the consultant secretaries and a link to the local MSK physiotherapy website, which provided self-directed rehabilitation resources. Patients were encouraged to initiate contact if their symptoms worsened or if they felt further clinical input was necessary. To minimise the risk of disengagement, patients with cognitive impairments, communication difficulties, or complex medical needs were excluded from the PIFU pathway.

### Data collection and statistical analysis

We reviewed clinical correspondence and electronic patient records for all eligible patients over the 18-month period. Key datapoints included the diagnosis at discharge, treatment received in clinic or referred for guided injection, whether patients re-accessed care within six months and the reason for return. We also identified which clinical conditions were most suited to PIFU and which were more likely to result in re-referral. Patients were considered to have re-accessed care if they returned to clinic via the PIFU process at any point during the study period. Additionally, we examined the GP records of patients who did not return to the clinic to confirm that they had not consulted their GP or another healthcare provider for the same condition.

Descriptive statistics were used to summarise patient outcomes, and qualitative patterns were explored to inform future PIFU optimisation.

## Results

**Figure 1:**
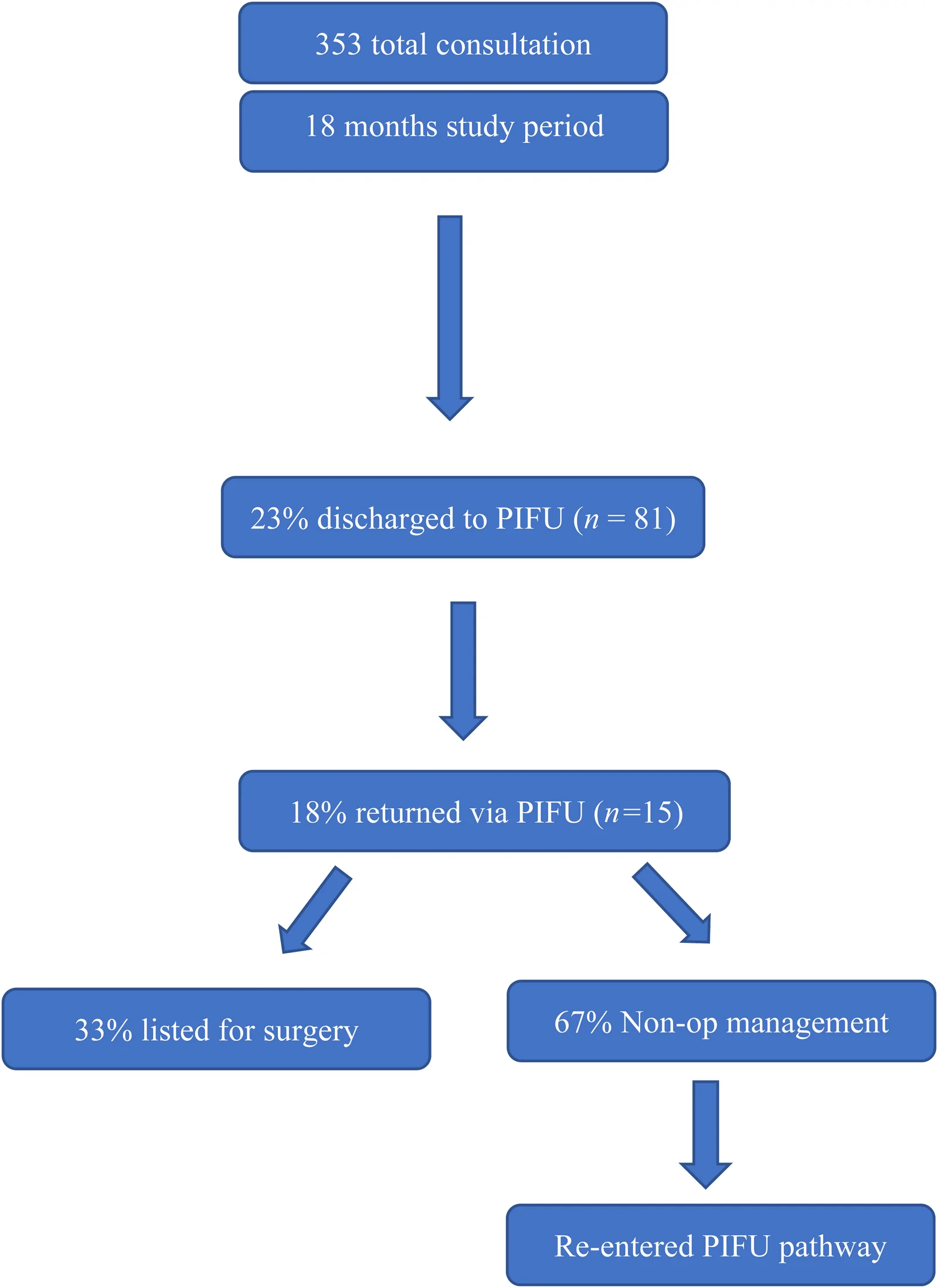
Flowchart: PIFU pathway PIFU = patient-initiated follow-up

A total of 353 consultations were reviewed over the 18-month study period. Key demographic details are summarised in Table 1. Of these, 81 patients (23%) were discharged under the PIFU pathway. Notably, 50 patients (62%) were first-time referrals discharged after their initial consultation, highlighting early adoption of PIFU in the care process (Figure 1). Referrals originated either directly from primary care or through local MSK triage services.

**Table 1:** Demographic data

Total number of PIFU	81
Average age, years	60
Sex, male:female	4:5
Appointments, new:follow-up	5:3
Average DASH score	49.6
Average EQ5D score	61.8

EQ5D = EuroQOL five dimensions questionnaire; DASH = disabilities of the arm, shoulder and hand questionnaire; PIFU = patient-initiated follow-up

### Diagnoses and interventions

The most frequent diagnoses among patients discharged to the PIFU pathway were (Table 2):

- Rotator cuff-related shoulder pain – 33%
- Glenohumeral osteoarthritis or cuff tear arthropathy – 19%
- Adhesive capsulitis (frozen shoulder) – 10%

**Table 2:** Diagnosis on discharge to PIFU

Total number	*n*=81
Rotator cuff-related shoulder pain	33%
Glenohumeral osteoarthritis or cuff tear arthropathy	19%
Adhesive capsulitis (frozen shoulder)	10%
Acromio-Clavicular joint arthritis	9%
Calcific tendinitis	7%

PIFU = patient-initiated follow-up

Of the 81 patients discharged to PIFU:

- 21 patients (26%) received corticosteroid and local anaesthetic injections during their clinic visit;
- 24 patients (30%) were referred for ultrasound-guided injections or hydrodialatation;
- 10 patients were referred to the pain team;
- 7 patients were noted to have resolving symptoms at discharge; and
- 10 patients were referred for physiotherapy.

### Re-presentations via PIFU

Within six months, 15 patients (18%) returned to the clinic through the PIFU process. This equated to only 4.2% of the total cohort, suggesting the pathway did not result in excessive or inappropriate re-attendance. While patients were eligible to re-access care for up to 12 months, only those with a minimum of 6 months’ follow-up opportunity were included in this analysis.

Of those who returned:

- 33% were listed for surgery, most commonly rotator cuff repair with or without subacromial decompression.
- The remaining patients received further injections and were re-entered into the PIFU pathway.

### Outcomes for nonreturning patients

For the 66 patients who did not re-access care, GP records were reviewed to determine whether they had sought treatment for the same or a similar upper limb problem elsewhere. This confirmed that none had re-presented to their GP or been referred to another clinician for the same condition during the follow-up period. These findings support both the safety and appropriateness of discharge via the PIFU pathway.

### Service impact and patient experience

The low re-presentation rate, combined with favourable clinical outcomes, suggests that PIFU is well suited to patients with stable or early-stage upper limb conditions, particularly where symptoms are mild to moderate and well controlled. The pathway empowers patients to manage their recovery independently, while maintaining a safety net for timely re-access if required. Importantly, the introduction of PIFU freed up approximately 20% of follow-up clinic slots, improving capacity for new and urgent referrals. Informal feedback indicated high levels of patient satisfaction, although more formalised patient-reported outcome measures are recommended for future evaluations.

## Discussion

This study provides valuable insights into the implementation and efficacy of PIFU in Orthopaedic Shoulder and Elbow Clinics, highlighting its potential to optimise outpatient care. As MSK conditions continue to rise, healthcare systems face increasing challenges in managing outpatient demand. Traditional clinician-driven follow-up models contribute to two-thirds of all outpatient visits, often leading to unnecessary clinic congestion and inefficient resource utilisation.^1^

Over an 18-month period, we assessed clinic outcomes and patient records to evaluate the feasibility and effectiveness of the PIFU model. A total of 353 consultations were reviewed, with 23% of patients discharged to PIFU, granting them the option to request follow-up appointments as needed. Notably, 50 of these were first-time GP referrals who were discharged after their initial consultation, indicating confidence in the early application of the PIFU pathway. The most common conditions among patients placed on PIFU included early glenohumeral osteoarthritis or cuff tear arthropathy, rotator cuff-related shoulder pain and adhesive capsulitis (frozen shoulder). Over half of this cohort (56%) received in-clinic injections or were referred for guided injection, highlighting that these conditions may be particularly suitable for nonoperative management and patient-led follow-up.

The high proportion of conservatively managed patients may reflect variability in triage efficiency and the thresholds applied for secondary care referral. Differences in the quality and completeness of referral information, as well as the clinical experience of triaging practitioners, can influence whether patients with conditions amenable to self-management or community-based care are directed to specialist clinics. In some cases, lower referral thresholds may lead to the inclusion of patients with milder symptoms or earlier-stage disease, who ultimately require only nonoperative interventions.

One of the key findings of this study was the return rate among PIFU patients. We observed that approximately 18% of PIFU patients returned to the clinic, representing just 4.2% of the total cohort. These figures indicate that PIFU does not significantly increase patient return rates, reinforcing its feasibility as a follow-up model. Additionally, by shifting the follow-up burden to patient-led appointments, nearly 20% of follow-up clinic slots were made available, potentially easing outpatient service pressures.

These findings align with existing research suggesting that PIFU models can reduce hospital reviews by 38% over six years without negatively impacting patient outcomes or satisfaction.^4^ Studies on rheumatoid arthritis, inflammatory bowel disease and breast cancer have demonstrated that PIFU does not compromise recurrence detection or patient-related outcomes, while also improving patient autonomy.^5^ In the context of orthopaedic care, PIFU offers a patient-centred approach, allowing patients to schedule follow-ups based on symptoms rather than routine appointments. This model not only enhances patient engagement but also optimises clinical time, ensuring that resources are allocated to those who need them most.^3^

However, despite its benefits, patient selection remains a critical factor in PIFU success. Conditions such as rotator cuff-related pain and frozen shoulder appear well-suited to PIFU, while others may require structured monitoring to prevent delayed interventions. Studies suggest that clear patient education and robust support mechanisms are essential for successful adoption.^6^ Additionally, healthcare accessibility and digital literacy play a crucial role in ensuring that patients can effectively engage with PIFU systems.^2^

Economic considerations also warrant further investigation. While PIFU has been associated with lower healthcare costs, including reduced hospital visits and travel expenses, its long-term financial impact on orthopaedic services remains unclear. A study on inflammatory bowel disease found that mean travel costs per patient were significantly lower in the PIFU group (£0.86 versus £8.92), demonstrating potential cost savings.^7^ Future research should focus on cost-effectiveness analyses, assessing whether PIFU can sustainably reduce outpatient expenditures while maintaining high-quality care.

## Limitations and future directions

This study has some limitations. The 18-month period may not fully capture long-term patient adherence and outcomes. A longer follow-up period would be helpful in understanding whether patients continue to engage with PIFU or require additional interventions. Additionally, since the study focused on a single orthopaedic clinic, the findings may not be generalisable to other healthcare settings. Future research should explore the broader adoption of PIFU across different orthopaedic centres.

Patient demographics and socioeconomic factors also play a role in PIFU success. Older patients and those with more complex conditions may be less likely to engage with PIFU, and therefore, tailored approaches may be necessary to ensure equitable access.^5^ Investigating digital health literacy, accessibility and patient preferences will be essential in refining PIFU strategies.

We were unable to collect additional data from patients who did not re-access the clinic, and therefore we cannot determine whether their nonreturn was due to symptom resolution, access barriers or other reasons. This represents a limitation of our study. Future work should incorporate structured patient feedback to better understand patient experiences and reasons for not re-engaging with the service.

## Conclusion

PIFU presents a promising alternative to traditional follow-up models, offering increased flexibility, enhanced patient autonomy and optimised clinical efficiency. However, more research is needed to evaluate its long-term effectiveness, condition-specific impact and economic viability in orthopaedic Shoulder and Elbow Clinics. By addressing these research gaps, healthcare systems can further refine PIFU strategies to ensure safe, effective and sustainable patient-centred care.
